# Wnt16 Is Associated with Age-Related Bone Loss and Estrogen Withdrawal in Murine Bone

**DOI:** 10.1371/journal.pone.0140260

**Published:** 2015-10-09

**Authors:** Henry Todd, Gabriel L. Galea, Lee B. Meakin, Peter J. Delisser, Lance E. Lanyon, Sara H. Windahl, Joanna S. Price

**Affiliations:** 1 School of Veterinary Sciences, University of Bristol, Bristol, United Kingdom; 2 Centre for Bone and Arthritis Research, Institute of Medicine, Sahlgrenska Academy at the University of Gothenburg, Gothenburg, Sweden; Institut de Génomique Fonctionnelle de Lyon, FRANCE

## Abstract

Genome Wide Association Studies suggest that Wnt16 is an important contributor to the mechanisms controlling bone mineral density, cortical thickness, bone strength and ultimately fracture risk. Wnt16 acts on osteoblasts and osteoclasts and, in cortical bone, is predominantly derived from osteoblasts. This led us to hypothesize that low bone mass would be associated with low levels of Wnt16 expression and that Wnt16 expression would be increased by anabolic factors, including mechanical loading. We therefore investigated Wnt16 expression in the context of ageing, mechanical loading and unloading, estrogen deficiency and replacement, and estrogen receptor α (ERα) depletion. Quantitative real time PCR showed that Wnt16 mRNA expression was lower in cortical bone and marrow of aged compared to young female mice. Neither increased nor decreased (by disuse) mechanical loading altered Wnt16 expression in young female mice, although Wnt16 expression was decreased following ovariectomy. Both 17β-estradiol and the Selective Estrogen Receptor Modulator Tamoxifen increased Wnt16 expression relative to ovariectomy. Wnt16 and ERβ expression were increased in female ERα^-/-^ mice when compared to Wild Type. We also addressed potential effects of gender on Wnt16 expression and while the expression was lower in the cortical bone of aged males as in females, it was higher in male bone marrow of aged mice compared to young. In the kidney, which we used as a non-bone reference tissue, Wnt16 expression was unaffected by age in either males or females. In summary, age, and its associated bone loss, is associated with low levels of Wnt16 expression whereas bone loss associated with disuse has no effect on Wnt16 expression. In the artificially loaded mouse tibia we observed no loading-related up-regulation of Wnt16 expression but provide evidence that its expression is influenced by estrogen receptor signaling. These findings suggest that while Wnt16 is not an obligatory contributor to regulation of bone mass per se, it potentially plays a role in influencing pathways associated with regulation of bone mass during ageing and estrogen withdrawal.

## Introduction

Wnt16 has emerged as a promising potential therapeutic target for osteoporosis as it is known to be associated with bone mineral density, cortical thickness, bone strength, and fracture risk [1–7]. It is one of a family of 19 secreted cysteine-rich glycoproteins that signal through the Wnt/Lrp5/Frizzled signaling system (extensively reviewed in [1]), and several human bone disorders have been connected to this pathway. Recently, Movérare-Skrtic et al reported that Wnt16 expression is higher in cortical bone than in other organs and that it is mainly expressed in osteoblasts in cortical bone but is not detectable in osteoclasts [8]. However, osteoblast-derived Wnt16 inhibits osteoclastogenesis indirectly by increasing osteoprotegrin and directly by acting on osteoclast progenitors [8]. As a consequence, targeted deletion of Wnt16 in osteoblasts, as well as global deletion of Wnt16, has been shown to lead to an increased number of osteoclasts, cortical thinning, and to a significant increase in the incidence of fractures both in male and female mice [8, 9].

Because Wnt16 clearly plays a role in regulating cortical bone mass this suggests that its expression may be regulated by mechanical loading. Bones are active organs continuously adapting their mass and architecture to the habitual loading to which they are exposed. Highly strenuous activities lead to increased bone mass; conversely, unloading, such as that induced by bed rest, leads to a rapid loss of bone. Ageing is also associated with a steady decline in bone mass and subsequently bone strength. This decline has been suggested to be attributed to, among others, reduced physical activity/loading of the skeleton as well as a reduced ability to respond to the loading placed upon it. The fact that many of the changes that occur in bone with advancing age are inducible by disuse has led our group, and others, to suggest that disuse may be a model for ageing [10, 11]. Interestingly Shen et al showed that Wnt16 expression in bone marrow does not appear to be regulated by ageing in humans [12].

In humans, ageing is associated with a reduction of several hormones including estrogen. Estrogen withdrawal is associated with an imbalance in bone remodeling in favor of bone resorption, leading to the accelerated bone loss observed at the menopause. However, the notion that the decrease in estrogen levels during ageing is associated with the more gradual loss of cortical bone observed with ageing has recently been challenged ([13] and references therein). Estrogen acts through its receptors (ERα and ERβ) to preserve bone. Several studies using ER deficient *in vivo* models have shown that ERα is the most important ER in bone of both genders and ERβ modifies ERα activity only in females [14–23]. We and others have demonstrated that ERα is necessary for, and ER β also affects, loading-induced cortical expansion in female, and possibly in male, mice [16, 21, 24–27].

Because loading and ER signaling play important roles in regulating cortical bone mass, this led us to explore the hypothesis that the low bone mass observed with ageing and disuse would be associated with low levels of Wnt16 expression and that its expression would be increased by mechanical loading and/or increased ER signaling. We thus investigated Wnt16 expression in the cortical bones of aged and young male and female mice, and in the context of mechanical loading and unloading, estrogen deficiency and replacement and estrogen receptor α (ERα) depletion.

## Material and Methods

### Animals

Female and male, young (16-week-old) and aged (19-month-old female and 22-month-old male) C57BL/6 mice (*n* = 6–10 per group) were obtained from Charles River Inc. (Margate, UK). All mice were allowed free access to water and a maintenance diet containing 0.75% calcium (EURodent Diet 22%; PMI Nutrition International, LLC, Brentwood, MO, USA) in a 12-hour light/dark cycle, with room temperature at 21 ± 2°C. Animals in the estradiol-ovariectomy (OVX) studies were kept on a phytoestrogen-free diet for two weeks before and during the entire experiment (R70, Lantmännen, Sweden). Peri-operative analgesia was provided by buprenorphine (Vetergesic, Alstoe, UK, 0.08mg/kg subcutaneously). The ERα depleted and Tamoxifen-treated mice were from previous studies from our laboratory [24, 28]. The mice were housed in groups of up to 5 animals and all cages contained wood shavings, bedding, and a cardboard tube for environmental enrichment. At the end of the experiments, the mice were sacrificed by anesthesia with ketamine (Vetalar, Zoetis, London, UK) and dexmedetomidine (Dexdomitor, Elanco, Basingstoke, UK), followed by exsanguination via cardiac puncture. Dislocation of the neck was performed to ensure death prior to dissection. All procedures complied with the UK Animals (Scientific Procedures) Act 1986 under a UK Government Home Office project license (PPL30/2829) and were reviewed and approved by the University of Bristol ethics committee (Bristol, UK).

### The Effect of Age on Wnt16 Expression

To determine Wnt16 expression in tissues of young and aged mice, male and female mice were sacrificed. Cortical bone, bone marrow and left kidneys were collected and immediately snap frozen in liquid nitrogen and later used for qRT-PCR.

### The effects of Mechanical Loading: Reduced Loading of the Tibia Induced by Sciatic Neurectomy

Reduction in habitual loading of the tibia on one side was achieved by unilateral sciatic neurectomy (SN). This was performed by resecting a 3- to 4-mm segment of the right sciatic nerve, posterior to the hip joint, under isoflurane-induced anesthesia. Mice underwent unilateral SN on day 1, and were sacrificed 3, 6, 12 or 24 hours, or two weeks later (day 15). Bilaterally tibial cortical bone and marrow were separated and immediately snap frozen in liquid nitrogen and later used for quantitative RT-PCR.

### The effects of Mechanical Loading: Increased Loading of the Tibia by External Mechanical Loading

The right tibias were subjected to a single period of external mechanical loading, under isoflurane-induced anesthesia, to investigate the effect of loading on Wnt16 expression. Left limbs were used as internal controls as previously validated [29, 30]. The protocol for non-invasively loading the mouse tibia has been reported previously [28, 30]. In brief, the flexed knee and ankle joints are positioned in concave cups; the upper cup, containing the knee, is attached to an actuator arm of a loading device and the lower cup to a dynamic load cell. The tibia is held in place by a 0.5N continuous static preload. Forty cycles of dynamic load are superimposed with 10-second rest intervals between each cycle. The protocol for one cycle consists of loading to the target peak load, hold for 0.05 seconds at the peak load, and unloading back to the 0.5N preload. All mice were allowed normal cage activity in between loading sessions. Following loading, mice were sacrificed after 1, 6, 12 or 24 hours and bilaterally, tibial cortical bone and marrow were separated and immediately snap frozen and later used for quantitative RT-PCR analyses.

### The Effects of Estradiol Treatment in Young Female Mice

Virgin female C57Bl/6 mice were sham-OVX (n = 10) or OVX (n = 20) at 16 weeks of age (day 1). Five days after the surgery (day 6) the OVX mice were randomly subdivided into two groups (n = 10). The sham-OVX and one OVX group were treated with 17β-estradiol-3-benzoate (E2, Sigma, Poole, UK) at either 0.5 or 10μg/mouse/day, with the remaining OVX group receiving vehicle (10% Molecular grade ethanol (Fisher, Loughborough, UK), 90% Miglyol 812 (Cremer Oleo, Witten, Germany)) by s.c. injection on days 6, 7, 8 and 9. Mice were sacrificed on day 10. Femurs were immediately snap frozen in liquid nitrogen and later used for quantitative RT-PCR.

### The Effects of Tamoxifen Treatment in Young Female Mice

Mice were treated with Tamoxifen (2 mg/kg/day) using a regimen that we have previously shown synergistically enhanced loading-related bone gain [28]. At 16 weeks of age (day 1), 16 virgin female C57BL/6 mice were OVX. Ten days after surgery (day 11), the OVX mice were randomly subdivided into two groups (n = 8) and received either vehicle (peanut oil, 5 ml/kg; Sigma) or tamoxifen citrate (Tocris Cookson Inc., Ellisville, MO) by s.c. injection on days 11, 13, 15, 18, and 21 and were then sacrificed on day 25. Tibias were immediately snap frozen in liquid nitrogen and later used for qRT-PCR.

### Quantitative Real-time PCR Analysis

For RNA extraction from bone, the surrounding muscle was dissected, the epiphyses were removed, and the marrow was removed by centrifugation in custom made bone holders. Bones were pulverized in QIAzol^TM^ using a TissueLyser LT^TM^ (Qiagen, Sussex, UK). RNA was extracted, and genomic DNA was eliminated using RNeasy^TM^ Plus Universal kits (Qiagen, Sussex, UK). First strand cDNA synthesis was performed using SuperScriptII^TM^ (Invitrogen, Paisley, UK). Quantitative real-time PCR was performed using the standard curve method with QuantiTect SYBR^®^Green (Qiagen, Germany) and a 7900HT Fast Real Time PCR system (Applied Biosystems). Samples were run in duplicates and the expression levels for all the genes analyzed were normalized relative to β2-microglobulin (β2-microglobulin). Average values were used for subsequent statistical analysis. PCR primers were retrieved from the Harvard Primer Bank as previously reported [31]. Primers were as follows: mouse *β2-microglobulin* sense ATGGCTCGCTCGGTGACCCT and anti-sense TTCTCCGGTGGGTG-CGTGA [32]; mouse *Wnt16* sense AGTGCAGGCAACATGACCG and anti-sense CCACATGCCGTACTGGAC ATC, mouse *Sost* sense GCCGCGAGCTGCACTACAC and anti-sense CACCACTTCACGCG CCCGAT [32]; mouse *ERβ (Esr2)* sense ACGGCTCTCT-ACATAGGAGGA and anti-sense GAGCTTCCCCGGGTGTCC; *EGR-2* sense GGCCAG-ACCAAGATCCAC and anti-sense AGCCCCCAGGAC CAGAGG [33]; *OPG* sense TGTGTGTCCCTTGCCCTGACCA and anti-sense ACACTCGGTTGTGGGTGCGG; *Axin2* sense ATGAGTAGCGCCGTGTTAGTG and anti-sense GGGCATAGGTTTGGTGGACT; and *Rankl* sense CAGCATCGCTCTGTTCCT GTA and anti-sense CTGCGTTTTCATGGAGTCTCA.

### Statistical analysis

Comparisons between two groups were by t-test following Levene’s test for homogeneity of variance. Comparisons between more than two groups were by analysis of variance with post-hoc Bonferroni correction in SPSS Statistics (v.17). Data is presented as the mean ± standard error and p < 0.05 was considered statistically significant.

## Results

### Wnt16 Expression in Bone is Reduced with Age in Female and Male mice

**Fig 1:**
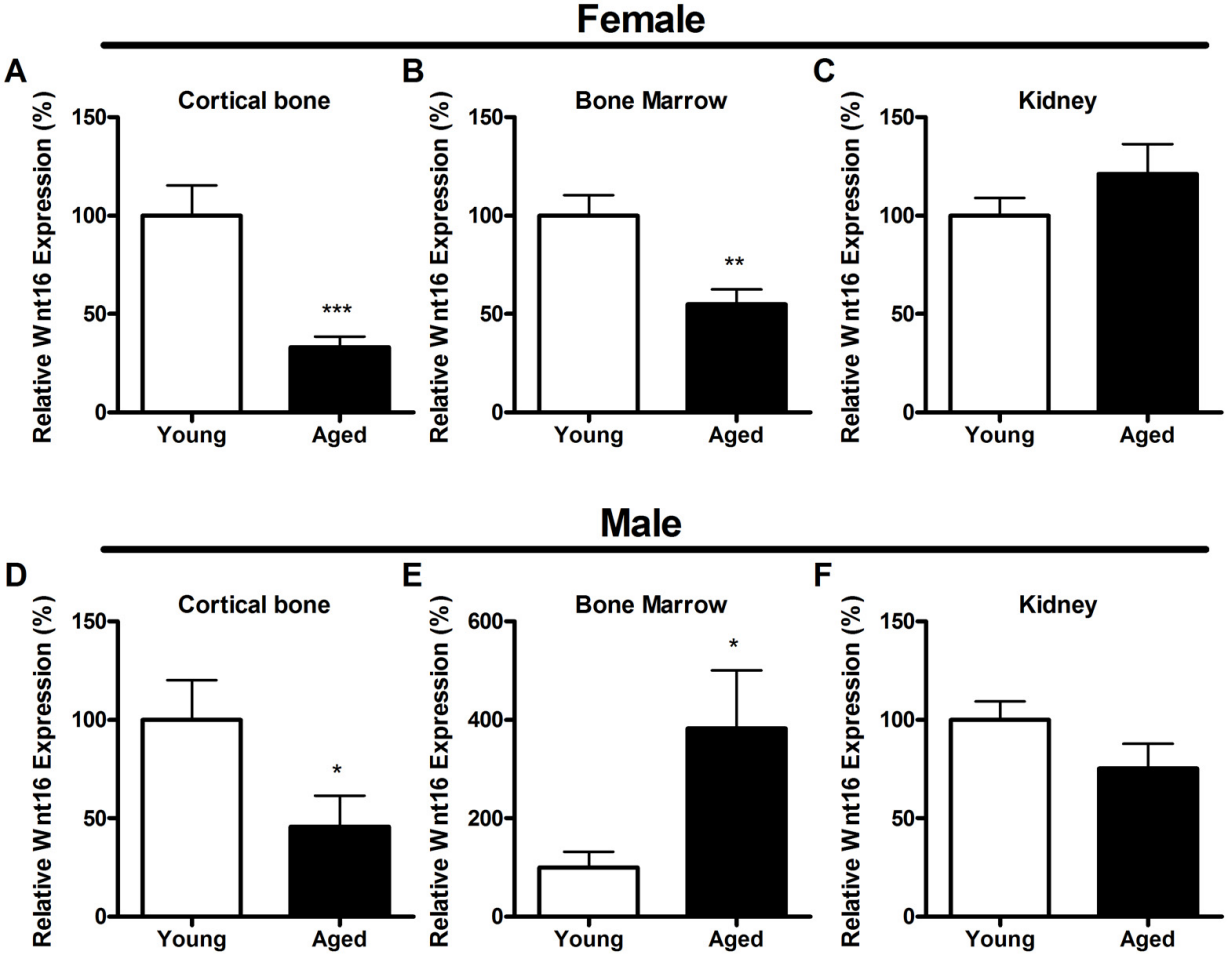
Wnt16 expression is decreased in femoral cortex of aged mice. Wnt16 expression was significantly lower in aged compared to young female (A) cortical bone and (B) bone marrow but unaffected by age in (C) kidney. Wnt16 expression was significantly lower in aged compared to young male (D) cortical bone and (E) bone marrow but unaffected by age in (F) kidney. Wnt16 expression was determined by quantitative RT-PCR and normalized relative to β2-microglobulin. Bars represent the mean ± SEM, * = p<0.05, ** = p<0.01 *** = p<0.001 vs. young females, N = 6–8.

We assessed Wnt16 expression in tissues from young and aged female femoral cortex, marrow and kidney (Fig 1A–1C). Wnt16 expression was significantly lower in cortical bone (-78%, p<0.001) and bone marrow (-45%, p<0.01) in aged compared to young mice. However, there was no difference in Wnt16 expression when comparing young and aged kidney. In order to investigate if the changes in Wnt16 expression during ageing are gender dependent, we also assessed Wnt16 expression in femoral cortex, marrow and kidney from young and aged male mice (Fig 1D–1F). In cortical bone Wnt16 expression was lower in aged than young males (-54%, p<0.05), but enhanced in male bone marrow (+282%, p<0.05). Wnt16 expression was not significantly different in young or old male compared to female cortical bone, although there was a non-significant trend towards a lower expression of Wnt16 in young male compared with young female cortical bone (-36%, p = 0.09). Although Wnt16 was expressed at a significantly lower level in both young and old male compared to female kidneys (-78% and -67%, respectively p<0.001), there was no difference in Wnt16 expression between kidneys from young and aged males (Fig 1F).

**Fig 2:**
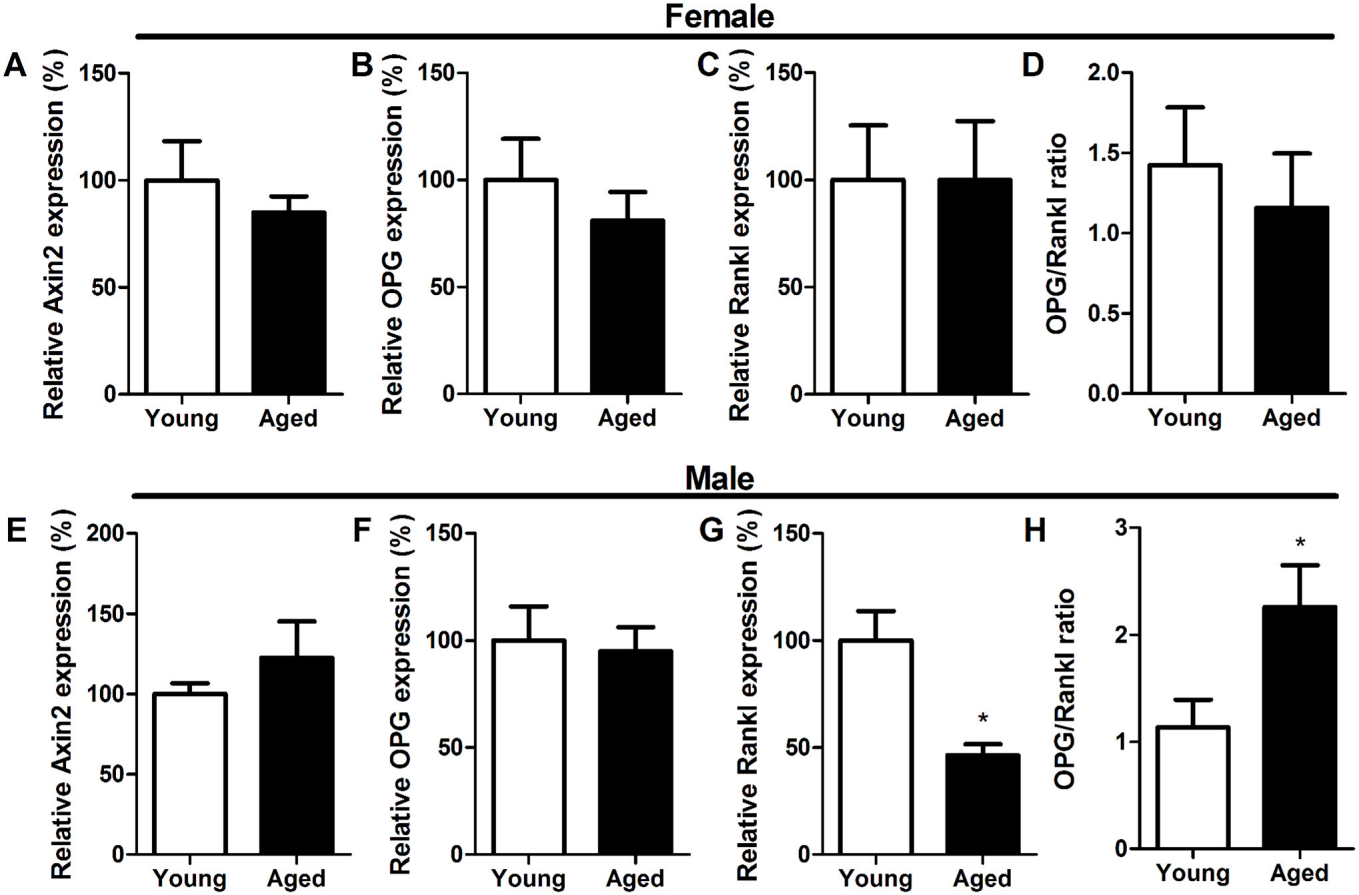
Ageing does not significantly alter expression of Axin2, OPG, or Rankl, in female mice but cortical bone Rankl expression is lower in aged than young male mice. (A,E) Axin2, (B,F) OPG, (C,G) Rankl and (D,H) the OPG:Rankl ratio were quantified in cortical bone from young and aged (A-D) female and (F-H) male mice by quantitative RT-PCR and normalized relative to β2-microglobulin. Bars represent the mean ± SEM, * = p<0.05, N = 8.

In females, the ageing-related reduction in Wnt16 expression was not associated with significant changes in the expression of the canonical Wnt targets Axin2 or osteoprotegerin (OPG) in cortical bone, nor of the OPG-binding regulator of osteoclast differentiation, receptor activator of nuclear factor κB ligand (Rankl) or the OPG:Rankl ratio (Fig 2A–2D). In male cortical bone, ageing was not associated with changes in Axin2 or OPG expression (Fig 2E and 2F), but aged male mice had lower Rankl expression resulting in a higher OPG:Rankl ratio than in young male mice (Fig 2G and 2H).

### Wnt16 Expression is Unaffected by Increased or Decreased Mechanical Loading

**Fig 3:**
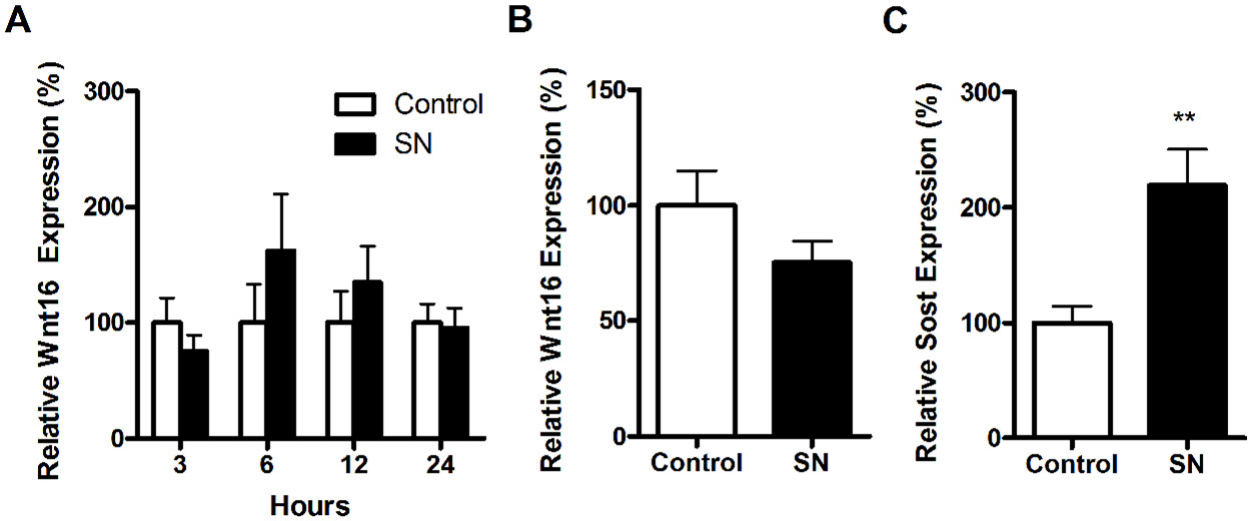
Wnt16 mRNA expression is not affected by unloading in young female mice. Wnt16 expression was unaffected by unloading for (A) 3, 6, 12 and 24 hours, and after (B) two weeks of unloading in young female mice. However, (C) Sost gene expression was significantly increased after two weeks of unloading. Gene expression was determined by quantitative RT-PCR and normalized relative to β2-microglobulin. Bars represent the mean ± SEM, ** = p<0.01 vs. sham operated control, N = 6. SN = sciatic neurectomy.

We analyzed the expression of Wnt16 in female mice subjected to disuse for 3, 6, 12 and 24 hours (Fig 3A) or 2 weeks (Fig 3B) in female mice. Disuse did not result in any significant changes in Wnt16 expression (Fig 3A and 3B). However, the expression of Sost, another regulator of the Wnt-pathway, was significantly increased (+118.6%, p = 0.004) after two weeks of disuse (Fig 3C). Sost expression was not significantly altered by disuse within the first 24 hours following neurectomy (data not shown).

**Fig 4:**
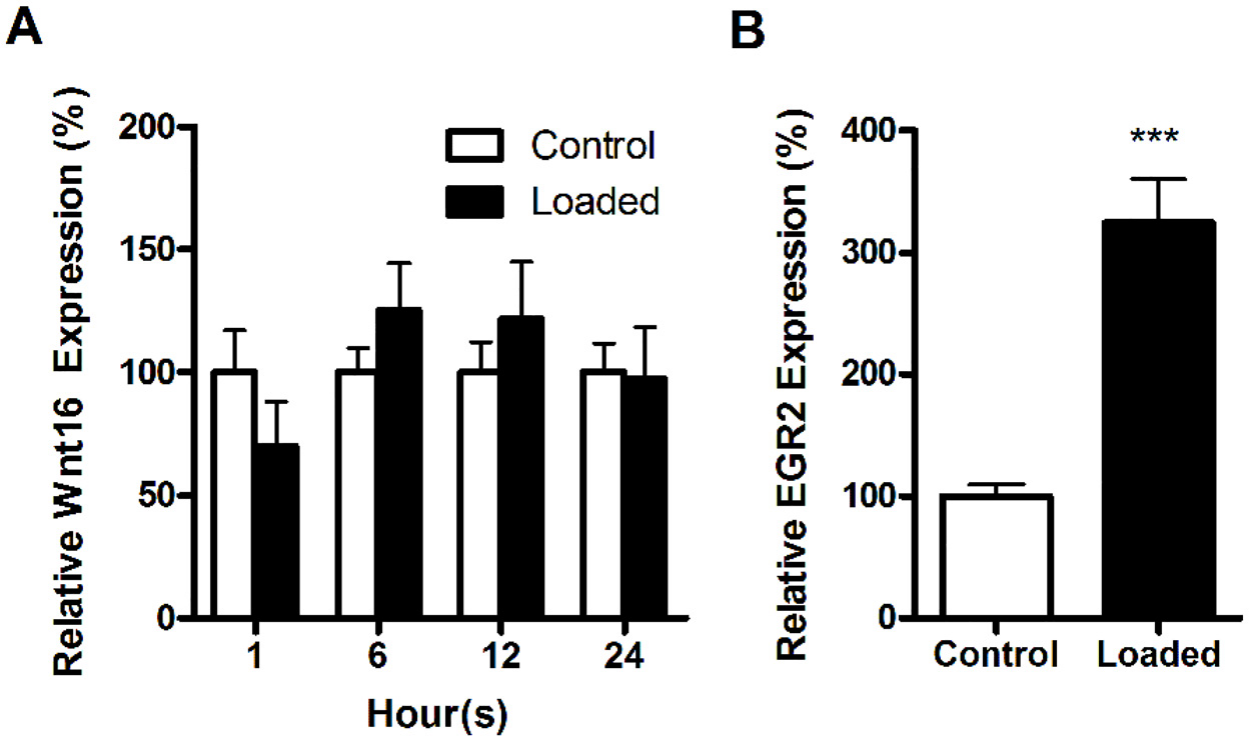
Wnt16 expression is not acutely affected by axial mechanical loading. (A) Wnt16 expression was unaffected 1, 6, 12 and 24 hours after a single episode of axial mechanical loading. However, (B) Egr2 expression was significantly up-regulated in young female mouse tibias after 1 hour. Gene expression were determined by quantitative RT-PCR and normalized relative to β2-microglobulin. Statistical analysis was by paired t-test. Bars represent the mean ± SEM. *** = p<0.001 vs. non-loaded control, N = 6–10.

We then investigated if Wnt16 could be involved in the anabolic effects of loading on cortical bone *in vivo*. Wnt16 expression was not altered after 1, 6, 12 or 24 hours of loading (Fig 4A). In contrast, the early growth response protein 2 (Egr2) was significantly up-regulated (+224%, p<0.001) 1 hour following a single period of loading (Fig 4B).

### Wnt16 Expression in Bones is Reduced by Ovariectomy and Enhanced by Estrogen Treatment

**Fig 5:**
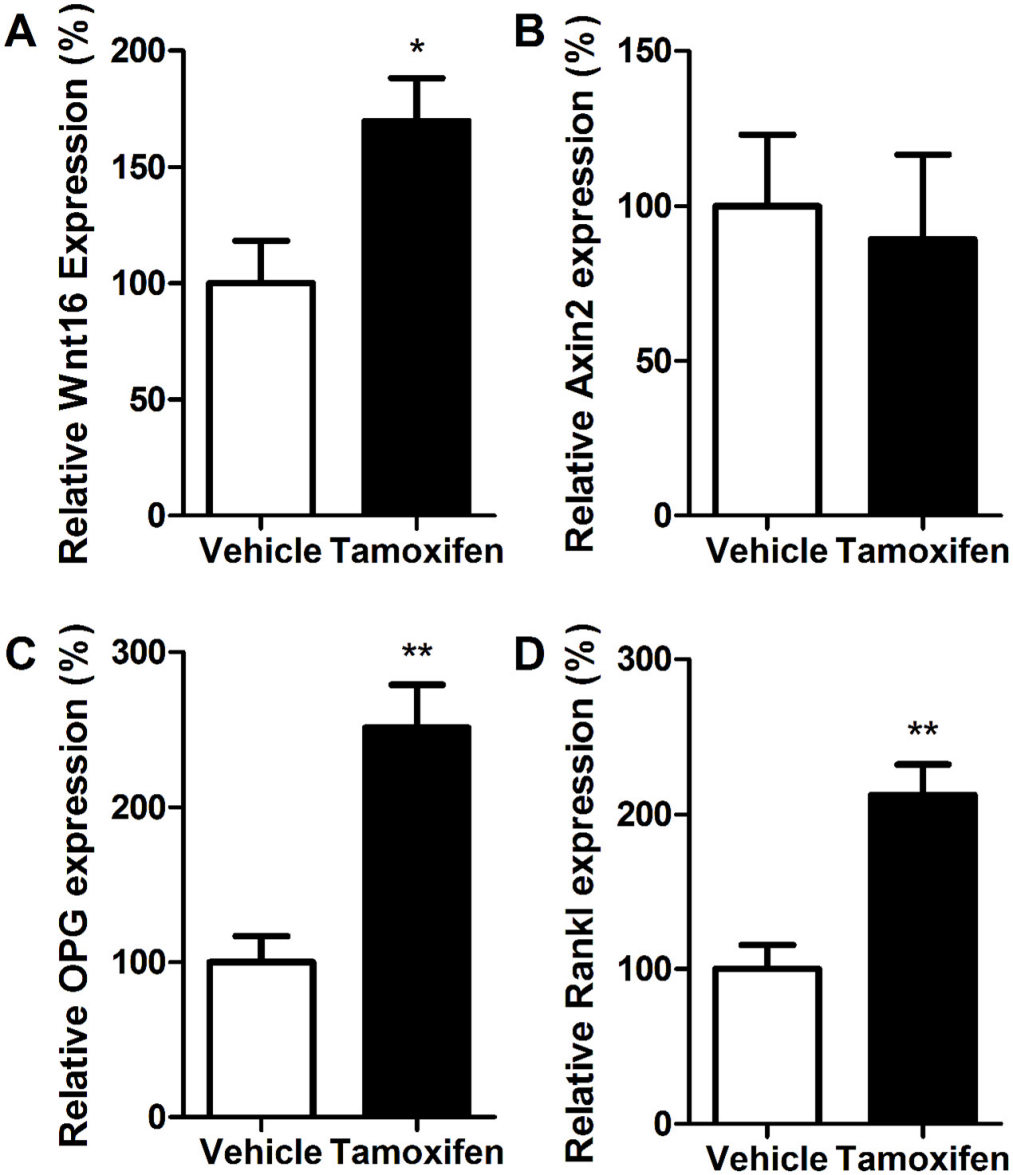
Tamoxifen treatment enhances Wnt16, Opg and Rankl expression in cortical bone of young female mice. (A) Wnt16, (B) Axin2, (C) OPG and (D) RANKL expression was quantified by qRT-PCR in ovariectomized young female mice treated with vehicle or tamoxifen. Bars represent the mean ± SEM. * = p<0.05, ** = p<0.01 vs. vehicle, N = 8.

We investigated Wnt16 expression in mice subjected to estrogen withdrawal following OVX or OVX with simultaneous treatment with the selective estrogen receptor modulator (SERM) Tamoxifen, or two doses of the endogenous estrogen 17β-estradiol (E2). Tamoxifen significantly increased Wnt16 expression by 70% (p<0.05) (Fig 5A), but was not associated with changes in Axin2 expression (Fig 5B). Tamoxifen significantly increased both OPG and Rankl (Fig 5C and 5D), such that the OPG:Rankl ratio was not significantly different between vehicle and tamoxifen-treated mice (p = 0.23, data not shown).

**Fig 6:**
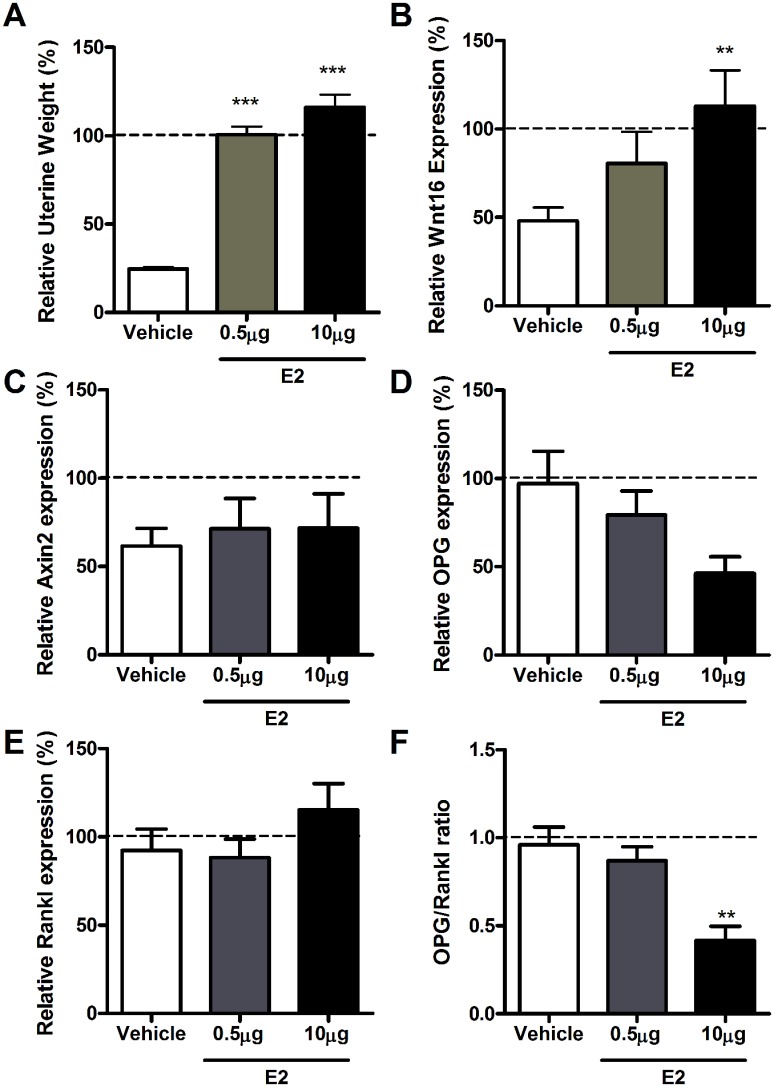
Estrogen treatment enhances Wnt16 expression in cortical bone of young female mice. (A) Both a low (gray bars) and a 20 times higher (black bars) dose of 17β-estradiol (E2) restored the uterus weight. (B) Wnt16, (C) Axin2, (D) OPG, (E) Rankl and (F) the OPG:Rankl ratio were determined by qRT-PCR in cortical bone from young female mice subjected to sham surgery or ovariectomized and then treated with vehicle, low dose or high dose of E2. Following qRT-PCR analysis, the sham-operated group was normalized to 100%, as indicated by the horizontal dashed line. Bars represent the mean ± SEM. ** = p<0.01,*** = p<0.001 vs. vehicle, N = 10.

Uterine weight decreased significantly in response to OVX (-75.4%, p<0.001 vs. sham) and was fully restored following E2 treatment (Fig 6A). Wnt16 expression was decreased by OVX (-51.9%; p<0.001 vs. sham), and was dose-dependently enhanced by 17β-estradiol in cortical bone (Fig 6B). These differences in Wnt16 expression were not associated with significant differences in Axin2 expression (Fig 6C). 17β-estradiol tended to down-regulate OPG expression (Fig 6D) without significantly changing Rankl (Fig 6E), resulting in a significant reduction in the OPG:Rankl ratio (Fig 6F) as has previously been reported [34].

**Fig 7:**
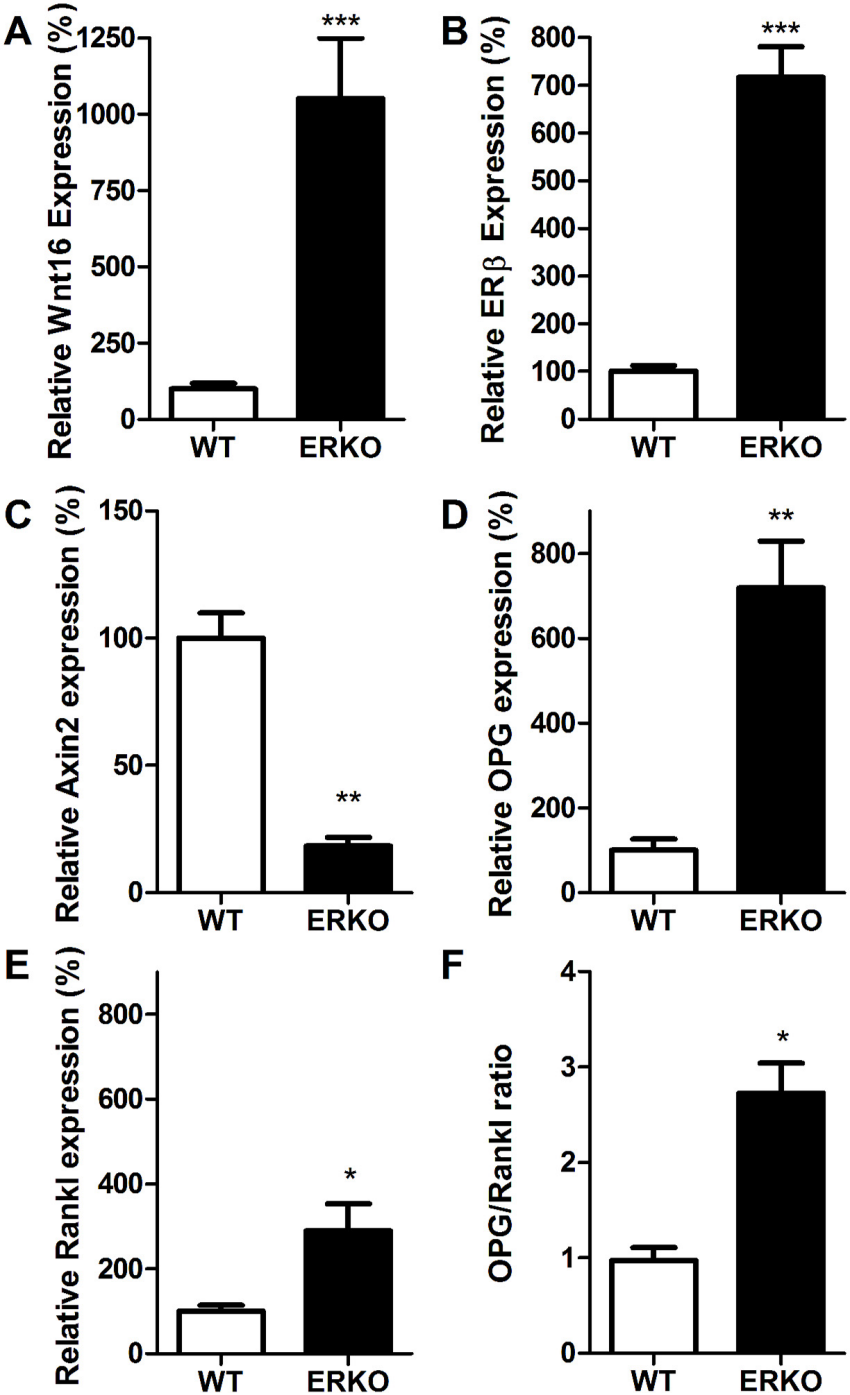
Wnt16 and ERβ expression are increased in cortical bone of ERα depleted female mice. (A) Wnt16, (B) ERβ, (C) Axin2, (D) OPG, (E) Rankl and (F) the OPG:Rankl ratio were quantified in young ERα depleted female mice and compared to WT mice. Gene expression was determined by quantitative RT-PCR and normalized relative to β2-microglobulin. Bars represent the mean ± SEM. * = p<0.05, ** = p<0.01,*** = p<0.001 vs. WT mice, N = 6.

We then analyzed Wnt16 and ERβ expression in ERα depleted female mice to study the involvement of the estrogen receptors in the regulation of Wnt16 expression. Both Wnt16 and ERβ expression were significantly increased in ERα depleted female mice (+952% and +616% respectively, p<0.001) (Fig 7A and 7B). In contrast to the females, neither Wnt16 nor ERβ expression were altered in male ERα depleted mice (data not shown). ERα depleted mice also had reduced expression of Axin2 (Fig 7C), increased OPG (Fig 7D, as previously reported in male ERα^-/-^ mice [34]), and increased Rankl (Fig 7E). The OPG:Rankl ratio was significantly higher in ERα^-/-^ than wild-type mice (Fig 7F).

## Discussion

Wnt16 has emerged as a promising therapeutic target, since it appears to be related to changes in bone mineral density, cortical thickness, bone strength and fracture risk. However, little is known about regulation of the Wnt16 gene itself. We hypothesized that since the effects of Wnt16 are primarily anabolic it would be down-regulated in conditions known to be associated with low bone mass and up-regulated in situations of increased bone mass. We therefore investigated the regulation of Wnt16 expression in the context of ageing, disuse, mechanical loading, estrogen deficiency and ER depletion in murine cortical bone. Our findings suggest that Wnt16 expression in bone and bone marrow, but not kidney, is gender-specifically altered with age and estrogen status. It is unaffected, however, by either increases or decreases in mechanical loading. This suggests that Wnt16 is not an obligatory contributor to increases in bone mass per se but is involved in some of the pathways, particularly those involving estrogen and age, which in turn result in alterations in bone mass.

It has recently been shown that cortical bone is the major source of Wnt16 in mice [8], although other Wnt ligands also play major roles in bone. Wnt1 mutations cause osteogenesis imperfecta in humans [35, 36], osteoblastic Wnt5a regulates osteoclastogenesis [37] at least in part through interaction with Wnt16 [38], overexpression of Wnt7b in osteoblasts dramatically increases bone mass [39], and Wnt4 overexpression in osteoblasts prevents bone loss associated with ovariectomy or ageing [40]. Here we report that differences in Wnt16 expression under different (re)modelling conditions are not directly linked to cortical bone expression of the canonical Wnt targets Axin2 and OPG, nor of the OPG interaction partner Rankl. Axin2 was not affected by any of the interventions tested except ERα deletion, which decreased Axin2 despite increasing Wnt16 expression. OPG was significantly increased by ERα deletion as have been previously described [34]and by treatment with the mixed ER agonist/antagonist tamoxifen, paralleling changes in Wnt16 expression in both situations, however short term estradiol treatment tended to down-regulate OPG resulting in a reduced OPG:Rankl ratio despite increasing Wnt16 expression. Rankl was up-regulated by ERα deletion as well as tamoxifen treatment, and was down-regulated by ageing in male but not female mice, again producing a pattern of differences in expression which does not directly mirror the differences observed in Wnt16. Further *in vitro* studies would be required to dissect out these potential interactions between Wnt16, estrogen receptor and downstream canonical Wnt/β-catenin signaling.

It has recently been shown that cortical bone is the major source of Wnt16 in mice, that it is essential for normal cortical bone thickness without altering trabecular bone mass [8,9] and that deletion of Wnt16 from osteoblasts, but not osteocytes, results in a dramatic increase in fracture risk [8]. As in humans, both male and female aged (19 month old) mice have reduced cortical thickness and reduced cortical as well as trabecular bone mass [11]. Our finding that Wnt16 is expressed at a lower level in cortical bone of aged compared to young mice of both genders could therefore be of significance, and provides a possible explanation to the cortical thinning and weakening of bones seen during ageing [41].”In a previous study, when a pool of male and female-derived human bone marrow from old individuals was compared with young [12], Wnt16 was not altered with age. In contrast here we show that Wnt16 expression in bone marrow is down-regulated in female but up-regulated in male bone marrow with age. This apparent discrepancy could be explained by the differences in species used (human vs. murine). Another explanation could be that the opposite responses on Wnt16-expression in male and female marrow were hidden when samples from the two genders were pooled in the previous study, but became apparent in our study where male and female marrow was compared separately between young and old groups.

The fact that many of the changes that occur in bone with advancing age are inducible by disuse has led our group, and others, to suggest that disuse may be a model for ageing [10, 11]. Two weeks of disuse, associated with increased Sost expression in the present study, reduces cortical bone mass and results in cortical thinning due to expansion of the medullary cavity in adult mice [42]. However, although Wnt16 was lower in aged bone compared to young bone, we did not observe any changes in Wnt16 expression either early (3–24 hours) or late (2 weeks) after the start of sciatic neurectomy-induced disuse. In contrast, we confirm previous results showing an increase in the Wnt inhibitor Sost two weeks after sciatic neurectomy [43]. This indicates that sciatic neurectomy induced a response in expression of other responsive genes in the Wnt-pathway. It is possible that in response to disuse, up-regulation of inhibitory proteins like Sclerostin is more important, whereas in ageing the regulation of the Wnt signaling molecules themselves appears affected. This suggests that ageing and disuse could be associated with bone loss through its effects on different targets within the same pathway.

Physical activity is the primary functional determinant of bone mass and architecture. It was recently shown using Wnt16 knockout mice that the anabolic effects of loading by 4-point bending is dependent on Wnt16 and that both axial loading and loading by four-point bending increase Wnt16 expression after two weeks of loading (at which point considerable new bone formation has occurred) [9]. The axial tibial loading protocol used in the present study when continued for two weeks, is potently osteogenic primarily due to periosteal modelling [44]. The initial cellular responses observed within the first 24 hours following loading include increased osteocyte and osteoblast metabolic activity [45], up-regulation of early response genes including Egr2, down-regulation of Sost/sclerostin [11, 33] and an increase in periosteal cell number [11] preceding new bone formation. However, we could not detect any alterations in Wnt16 expression during the first 24 hours after axial loading, although we could reproduce previous results showing an increase in EGR2 expression 1 hour after loading indicating that the strain applied was sufficient to engender a biological response [25, 33]. One possible explanation for the apparent contradictory results is that Wnt16 is not a primary target during mechanotransduction that leads to the acute increase in osteoblast number and/or activity. We have shown that the greatest number of adaptive transcriptomic changes in response to loading occur within the first 3 hours [33] and increases in periosteal osteoblast number are evident within 24 hours [11]. These responses occur before changes in Wnt16 expression are detected. However, Wnt16 may act later in the process of mechanotransduction, either through its upregulation or through down-regulation of its antagonists including sclerostin, thereby influencing the osteogenic context in which subsequent (re)modelling stimuli act.

We have previously reported that the ERs influence both the acute responses of bone to mechanical strain and the cellular context in which these stimuli act. There are several putative estrogen response elements half-sites in the Wnt16 promoter and recently it was reported that the Wnt16-promoter also contains a functional c-Jun binding site [46], indicating that estrogen and/or ERs could directly and/or indirectly regulate the Wnt16 promoter. Indeed, Wnt16 expression was reduced in response to ovariectomy-induced estrogen withdrawal, which if maintained for several weeks reduces cortical bone mass in mice as in humans [10, 28]. Wnt16 expression was normalized by E2-treatment, supporting a direct regulation of the Wnt16 gene by estrogens. Furthermore, Wnt16 expression was increased by tamoxifen which acts as a mixed ER agonist/antagonist. We have previously reported that tamoxifen administered to ovariectomised mice following the same protocol used in this study down-regulates Sost expression [47], increases cortical and trabecular bone mass and synergistically enhances the osteogenic response to loading.

Wnt16 expression was normalized by E2-treatment and enhanced by tamoxifen treatment, supporting a direct regulation of the Wnt16 gene by estrogens. In addition, Wnt16 expression was significantly higher in cortical bone of ERα-depleted female mice that have 10-fold higher levels of estrogen than WT mice [15, 18]. Adult female ERα-depleted mice have increased trabecular bone but reduced cortical area and a diminished cortical osteogenic response to mechanical loading [25, 48]. Our finding, that ERβ expression is also high in cortical bone of these mice, indicates that ERβ activated by high estrogen-levels could act on the Wnt16 promoter in cortical bone to compensate for the loss of ERα in female mice. These findings could also, at least in part, explain the difference in bone mass between estrogen-depleted mice (where no ligand-dependent ER activity is present) that have very low bone mass, and ERα depleted mice (where ERβ is activated) that have only slightly reduced bone mass. Because estrogen is one of the hormones that is reduced with age, and the ERs themselves are regulated by estrogens in bone [26], it is possible that the down-regulation of Wnt16 with age could be due to decreased estrogen signaling.

In summary, we have shown that in bone and bone marrow, but not kidney, Wnt16 expression is regulated in a gender-specific manner with age. It is down-regulated in female cortical bone and bone marrow, while it is down-regulated in male cortical bone and up-regulated in male bone marrow. Wnt16 expression is up-regulated in cortical bone by estrogen and the SERM Tamoxifen, but not by disuse or increased mechanical loading. This suggests involvement of Wnt16 in some but not all of the pathways participating in the regulation of bone mass.
